# Advances in Epigenetic Cancer Therapeutics

**DOI:** 10.7759/cureus.11725

**Published:** 2020-11-27

**Authors:** Christopher Hillyar, Kathrine S Rallis, Jajini Varghese

**Affiliations:** 1 Oncology, Barts and the London School of Medicine and Dentistry, Queen Mary University of London, London, GBR; 2 Breast and Plastic Surgery, University College London Institute of Surgery and Interventional Science & Royal Free NHS Trust, London, GBR

**Keywords:** cancer, epigenetics, dna methylation, histone modifications, inhibitors, histone methyltransferase, histone demethylase, histone acetyltransferase, histone deacetylase

## Abstract

Cancer has traditionally been hailed a genetic disease, dictated by successive genetic aberrations which alter gene expression. Yet, recent advances in molecular sequencing technologies, enabling the characterisation of cancer patient phenotypes on a large scale, have highlighted epigenetic changes as a hallmark of cancer. Epigenetic modifications, including DNA methylation and demethylation and histone modifications, have been found to play a key role in the pathogenesis of a wide variety of cancers through the regulation of chromatin state, gene expression and other nuclear events. Targeting epigenetic aberrations offers remarkable promise as a potential anti-cancer therapy given the reversible nature of epigenetic changes. Hence, epigenetic therapy has emerged as a rapidly advancing field of cancer research. A plethora of epigenetic therapies which inhibit enzymes of post-translational histone modifications, so-called ‘writers’, ‘erasers’ and ‘readers’, have been developed, with several epigenetic inhibitor agents approved for use in routine clinical practice. Epigenetic therapeutics inhibit the methylation or demethylation and acetylation or deacetylation of DNA and histone proteins. Their targets include writers (DNA methyltransferases [DNMT], histone acetyltransferases [HAT] and histone deacetylases [HDAC]) and erasers (histone demethylases [HDM] and histone methylases [HMT]). With new epigenetic mechanisms increasingly being elucidated, a vast array of targets and therapeutics have been brought to the fore. This review discusses recent advances in cancer epigenetics with a focus on molecular targets and mechanisms of action of epigenetic cancer therapeutics.

## Introduction and background

**Figure 1:**
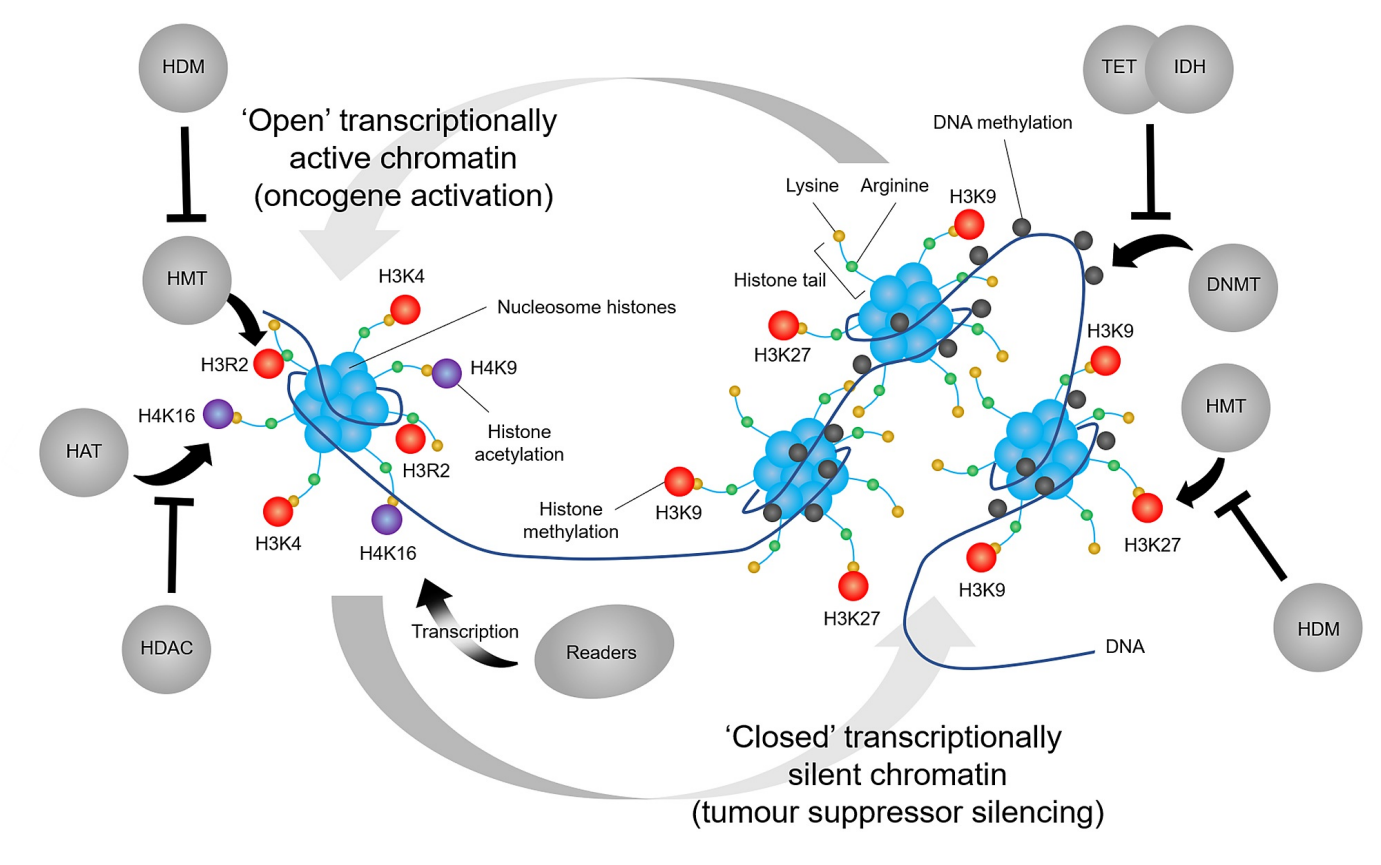
The antagonistic role of writers and erasers changes chromatin structure and regulates gene transcription, activating and silencing oncogenes and tumour suppressors In the nucleus, the human genome is highly organised into a tightly packaged DNA-protein complex called chromatin, which consists of basic units called nucleosomes. Nucleosomes comprise 147 base-pairs of DNA wrapped around an octamer of histone proteins (two H2A-H2B dimers and one H3-H4 tetramer), which is covalently modified by enzymes called ‘writers’, ‘erasers’ and ‘readers’. These epigenetic machines, which are the target of different anti-cancer drugs, are responsible for maintaining, removing and binding to epigenetic modifications to direct epigenetic effects. Certain families of writers and erasers act antagonistically to orchestrate changes in transcriptionally active euchromatin and transcriptionally silent heterochromatin Adapted from [3]. DNMT, DNA methyltransferase; HAT, histone acetyltransferase; HDAC, histone deacetylase; HDM, histone demethylase; HDMI, histone demethylase inhibitor; IDH, isocitrate dehydrogenase; TET, ten-eleven translocation protein

Cancer is a leading cause of avoidable premature death in the United Kingdom (UK) [1]. One in two people born after 1960 can expect to be diagnosed with cancer in their lifetime, with 367,167 new cancer cases and 164,901 cancer deaths occurring nationally each year in the UK [2]. Although risk factors vary between cancer types, cancer is primarily a genetic disease arising through successive genetic aberrations. Through projects such as the International Cancer Genome Consortium and The Cancer Genome Atlas Program, the consequences of cancer gene mutations are emerging. However, the role of the epigenome in reshaping gene expression profiles has also come to light (Figure 1). This review aims to highlight recent advances in our understanding of cancer epigenetics and the key targets for and mechanisms of epigenetic cancer therapeutics.

## Review

Table 1 provides a list of select epigenetic cancer therapeutics under development. Despite advances in cancer epigenetics, only two epigenetic cancer therapeutics are currently recommended for the treatment of cancer patients by the National Institute of Health and Care Excellence (NICE) in the UK. These include the DNA methyltransferase (DNMT) inhibitor 5′-azacytidine (Aza; Vidaza®) for myelodysplastic syndrome and certain types of leukaemia [4], and the histone deacetylase (HDAC) inhibitor panobinostat (LBH589) for multiple myeloma [5]. In addition, the enhancer of zeste homolog 2 (EZH2) inhibitor tazemetostat (EPZ-6348) has been granted orphan drug approval in Europe for large B-cell lymphoma by the European Medicines Agency [6]. In the United States, however, nine epigenetic therapeutics have been approved by the U.S. Food and Drug Administration, including two DNMT inhibitors (DNMTIs), four HDAC inhibitors, two isocitrate dehydrogenase (IDH) inhibitors, and, recently, the EZH2 inhibitor tazemetostat [7].

**Table 1:** Select epigenetic cancer therapeutics Adapted from [8]. DNMT, DNA methyltransferase; HAT, histone acetyltransferase; HDAC, histone deacetylase; HDM, histone demethylase; HMT, histone methyltransferase; IDH, isocitrate dehydrogenase; DNMT1, DNA (cytosine-5)-methyltransferase 1; DNMT3, DNA (cytosine-5)-methyltransferase 3; p300, E1A binding protein p300; CBP, CREB-binding protein; PCAF, P300/CBP-associated factor; LSD1, lysine-specific histone demethylase 1A; JMJD3, histone H3 lysine 27 (H3K27) demethylase KDM6B; KDM5B, lysine-specific demethylase 5B (also known as histone demethylase JARID1B); DOT1L, histone H3K79 methyltransferase; EZH1, enhancer of zeste homolog 1 (histone-lysine N-methyltransferase); EZH2, enhancer of zeste homolog 2 (histone-lysine N-methyltransferase);  G9a, protein-lysine methyltransferase; PRMT1/3/4/5/6/8, protein arginine N-methyltransferase 1/3/4/5/6/8; SUV39H1, histone-lysine N-methyltransferase

Group	Target	Compound	Cancer
DNMT inhibitor	DNMT1	5-aza-2′-deoxycytidine (5-aza-CdR; decitabine, Dacogen®)	Myelodysplastic syndrome, acute myeloid leukaemia
	DNMT1	5-azacytidine (5-aza-CR; Aza; Vidaza®)	Myelodysplastic syndrome, acute myeloid leukaemia
	DNMT1, DNMT3, cytidine deaminase	Zebularine (NSC309132; 4-deoxyuridine)	Hematologic and solid cancers
	DNMT1	Guadecitabine (SGI-110)	Hematologic and solid cancers
HAT inhibitor	p300	C646	Prostate cancer
	p300, CBP	Curcumin	Multiple myeloma, breast cancer, pancreatic cancer
	p300, PCAF	Anacardic acid	NA
	p300, PCAF	Garcinol	NA
HDAC inhibitor	Class I, II, IV HDAC	Sulforaphane (SFN)	Leukaemia, colorectal cancer, prostate cancer, other solid tumours
	Class I HDAC	Domatinostat (4SC-202)	Leukaemia, colorectal cancer
	Class I, II, IV HDAC	Resminostat (4SC-201, RAS2410)	Leukaemia, colorectal cancer, head and neck cancer, hepatocellular carcinoma
	Class I, II, IV HDAC	Panobinostat (LBH589)	Cutaneous T-cell lymphoma, Hodgkin’s lymphoma, breast cancer, head and neck cancer, prostate cancer, colorectal cancer, thyroid cancer
	Class I, II, IV HDAC	Vorinostat (SAHA, Zolinza®)	Cutaneous T-cell lymphoma, leukaemia, prostate cancer, bladder cancer, breast cancer
	Class I, II, IV HDAC	Romidepsin (depsipeptide, FK228)	Cutaneous T-cell lymphoma
	Class I HDAC1, 9, 11	Entinostat (MS-275, SNDX-275)	Hodgkin lymphoma, kidney cancer, breast cancer
	Class I, IV HDAC	Mocetinostat (MGCD0103)	Follicular lymphoma, Hodgkin's lymphoma and acute myelogenous leukaemia, chronic lymphocytic leukaemia, myelodysplastic syndrome, solid cancers
	Class I, II, IV HDAC	Pracinostat (SB939)	Myelodysplastic syndrome, acute myeloid leukaemia
	Class I, II, IV HDAC	Belinostat (PXD101)	Leukaemia, colorectal cancer, lung cancer, pancreatic cancer
HDM inhibitor	HDAC-LSD1	4SC-202	Hematologic malignancies
	JmjC domain proteins	GSK-J1, GSK-J4	NA
	JMJD3	GSK-J1	NA
	KDM5B	EPT-103182	Hematologic and solid cancers
	LSD1	GSK2879552	Acute myeloid leukaemia, small cell lung cancer
	LSD1	GSK354, GSK690	Acute myeloid leukaemia
	LSD1	NCD25, NCD38	Myelodysplastic syndrome
	LSD1	ORY-1001	Acute leukaemia
	LSD1	Tranylcypromine	Myelodysplastic syndrome, acute myeloid leukaemia
HMT inhibitor	DOT1L	EPZ00477	Hematologic malignancies
	DOT1L	Pinometostat (EPZ-5676)	Hematologic malignancies
	DOT1L	SGC0946	Leukaemia
	EZH1	UNC1999	Diffuse large B-cell lymphoma
	EZH2	3-Deazaneplanocin A (DZnep)	Acute myeloid leukaemia
	EZH2	GSK126	B-cell lymphoma
	EZH2	Tazemetostat (EPZ-6348)	B-cell lymphoma
	EZH2	EI1	Diffuse large B-cell lymphoma
	EZH2	EPZ005687	EZH2 mutant non-Hodgkin lymphoma
	EZH2	GSK343	Ovarian
	EZH2	DZNep	Breast, colon, prostate
	G9a	BRD4770	Pancreatic
	G9a	UNC0638	Acute myeloid leukaemia, breast
	G9a	BIX-01294	Leukaemia, bladder
	PRMT1	AMI-408	Acute myeloid leukaemia
	PRMT1/3/4/6/8	MS023	NA
	PRMT5	GSK3326595	Solid tumours, non-Hodgkin lymphoma
	SUV39H1	Chaetocin	Lymphomas, leukaemia, colorectal cancer, lung cancer
IDH inhibitor	IDH1	Ivosidenib (AG-120; Tibsovo®)	Acute myeloid leukaemia, myelodysplastic syndrome
	IDH2	Enasidenib (AG-221; Idhifa®)	Acute myeloid leukaemia, myelodysplastic syndrome

Therapeutics targeting DNA methylation and demethylation

The writers and erasers responsible for regulating DNA methylation include DNMTs and ten-eleven translocation (TET) proteins. DNMTs (DNMT1/2/3) transfer methyl groups from the methyl donor S-adenosyl methionine (SAM) to the 5’ position of cytosine, forming 5-methylcytosine (5mC). Although deposition of 5mC in the gene promoter is recognised as a cause of gene repression, gene activation may result from 5mC deposition in hypermethylated promoters and enhancer elements [9]. Generally, however, DNA methylation of ‘CpG islands’ (CGIs), which are highly concentrated clusters of cytosine-phosphate-guanosine (CpG) dinucleotides, restricts binding of transcription factors at promoters, while promoter CGI hypomethylation allows transcription factor binding and gene activation.

**Figure 2:**
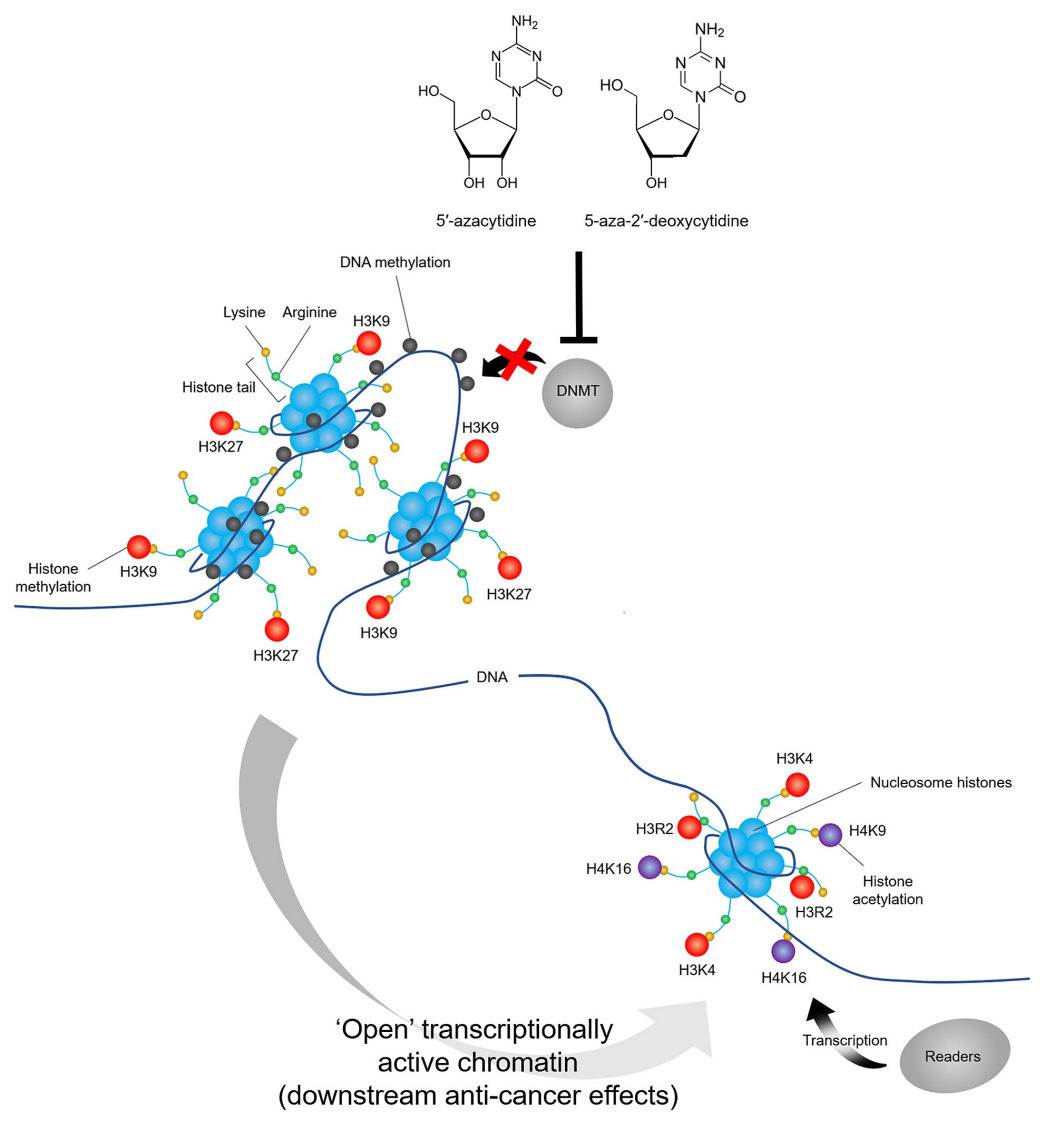
Mechanism of action of DNA methyltransferase inhibitors 5′-azacytidine (Aza; Vidaza) and 5-aza-2′-deoxycytidine (decitabine; Dacogen) incorporate into DNA and irreversibly bind to DNMT1, leading to DNA-DNMT1 adduct formation, DNMT1 degradation and, consequently, DNA demethylation and thus leading to the re-expression of tumour suppressor genes with downstream anti-cancer effects. DNMT1, DNA methyltranferase-1

Dysregulation of these processes occurs in cancer. A number of anti-cancer drugs have been developed that target DNMTs. DNMTIs, such as the cytosine analogues Aza (Vidaza) and 5-aza-2′-deoxycytidine (decitabine; Dacogen®), and the second-generation hypomethylating prodrug SGI-110 (guadecitabine) are classed as DNA hypomethylating agents. Their mechanism of action involves incorporation into DNA and irreversible binding to DNMT1, leading to DNA-DNMT1 adduct formation, DNMT1 degradation and, consequently, DNA demethylation (Figure 2). Aza also incorporates into RNA, more efficiently than DNA, following phosphorylation by uridine-cytidine kinase into triphosphates, resulting in polyribosome disassembly, defective methylation and acceptor function of transfer RNA and translation inhibition [10,11]. DNMTIs reduce aberrant hypomethylation and reactivate of silenced genes, thus restoring the function of tumour suppressor genes and DNA repair genes. In addition to the reactivation of tumour suppressor genes, DNMTIs enhance tumour immunogenicity through the upregulation of major histocompatibility complex (MHC) class I, leading to the recruitment of macrophages, natural killer (NK) cells and CD8+ T cells that secrete a variety of chemotactic and cytotoxic cytokines [12].

In contrast to DNMTs, erasers, such as TET proteins, demethylate DNA. TET proteins include a family of enzymes (TET1/2/3) that utilise Fe(II) and 2-oxoglutarate (alpha-ketoglutarate) as cofactors to oxidise 5mC to 5-hydroxymethylcytosine (5hmC). 5hmC formation alters transcriptional activation of gene expression. TET proteins also convert 5hmC to 5-formylcytosine (5fC) or 5-carboxylcytosine (5caC) before an enzyme called thymine-DNA glycosylase excises 5fC and 5caC from DNA. TET protein function is supported by IDH enzymes, which provide the essential 2-oxoglutarate cofactors through conversion of isocitrate to 2-oxoglutarate, and, together, these mechanisms complete DNA demethylation [13].

**Figure 3:**
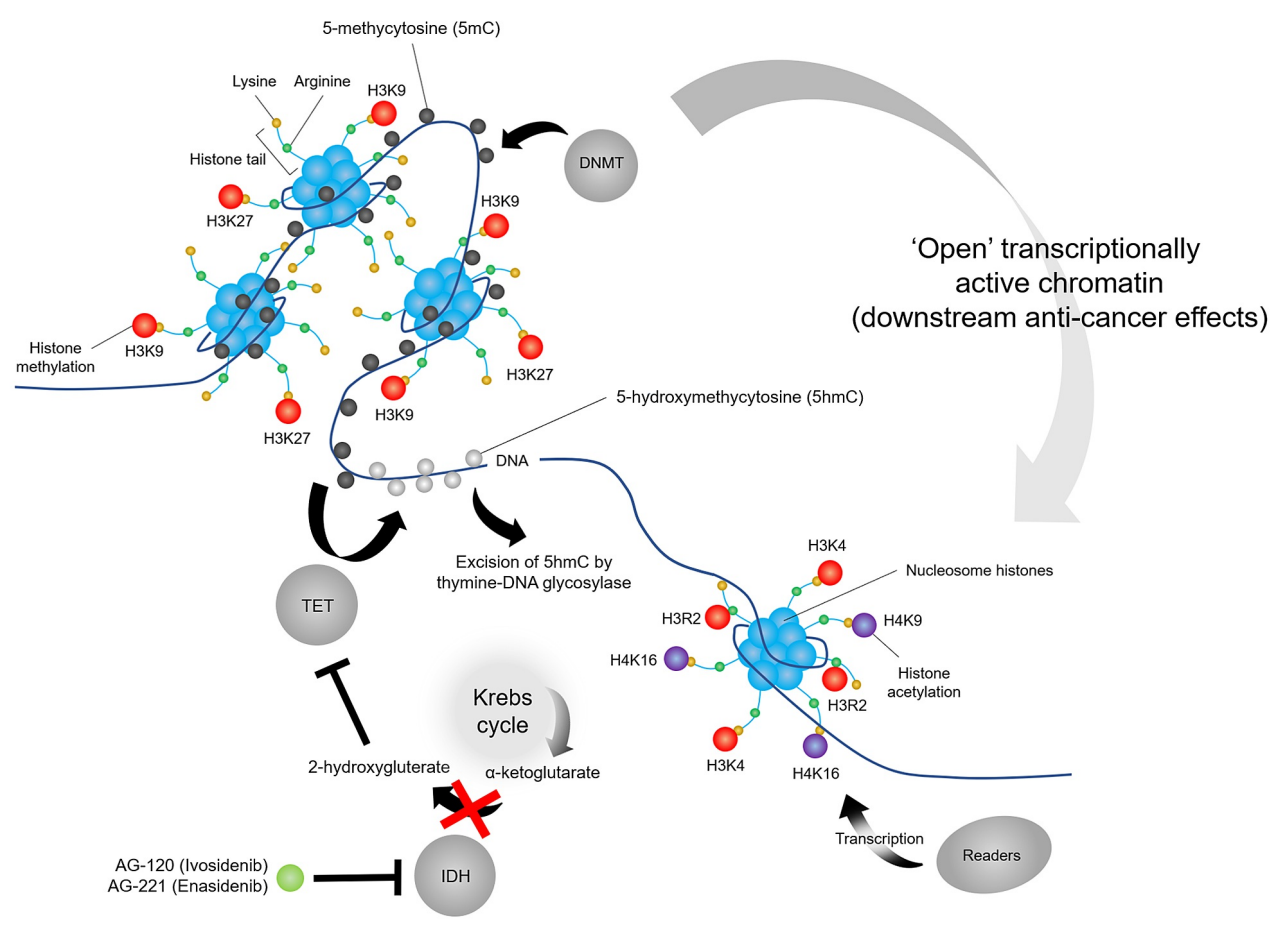
Mechanism of action of IDH inhibitors DNMT enzymes methylate cytosine bases (5-methylcytosine [5mC]), while TET proteins sequentially oxidise 5mC to 5-hydroxymethylcytosine (5hmC), which is ultimately excised from DNA, leading to DNA hypomethylation. IDH1/2 enzymes convert α-ketoglutarate to 2-hydroxygluterate, with the latter inhibiting the function of TET enzymes. IDH inhibitors AG-120 (ivosidenib) and AG-221 (enasidenib) reduce the accumulation of 2-hydroxygluterate, relieving inhibition of TET-dependent DNA demethylation, leading to downstream anti-cancer effects. DNMT, DNA methyltransferase; TET, ten-eleven translocation; IDH, isocitrate dehydrogenase

IDH1 and IDH2 mutations are present across several cancers including in 5-16% and 6-19% of acute myeloid leukaemia (AML), respectively [14]. TET2 loss-of-function mutations are present in 16% of AML [15]. Anticancer drugs that alter IDH and TET enzyme activity have been developed, including the IDH inhibitors AG-120 (ivosidenib) and AG-221 (enasidenib). These drugs inhibit IDH1/2-mediated conversion of α-ketoglutarate to 2-hydroxygluterate [16]. 2-hydroxygluterate is an oncometabolite that competitively inhibits TET enzymes [17], which leads to loss of 5hmC and is associated with carcinogenesis across several malignancies (Figure 3) [18]. IDH inhibitors induce primary AML cell differentiation [19]. In clinical trials, ivosidenib induced durable remission [20], while enasidenib was found to be well tolerated and induced responses in AML patients [21].

Therapeutics targeting modifications in histone tails

Writers responsible for covalent modifications on charged NH2 termini (tails) of histones include histone acetyltransferases (HATs; GCN5, MYST, p300/CBP families) and histone methyltransferases (HMTs; SET [(Su(var)3-9, enhancer of zeste, trithorax)] domain containing and non-SET-domain containing lysine-specific and arginine-specific families). In addition to acetylation and methylation, histone modifications include phosphorylation, ubiquitylation, sumoylation and biotinylation. The type and location of these modifications alters chromatin structure and gene expression. Gene-activating histone modifications include acetylation of lysine 27 in histone H3, methylation of lysines 4 and 36 in histone H3, and demethylation of lysine 9 of histone H3. Transcriptionally repressive histone modifications include methylation of lysine 9 and 27 in histone H3 and sumoylation of lysine 59 in histone H4.

Some histone modifications (H3K27 methylation) form docking sites for interactions with polycomb group proteins (PcGs). Once docked, polycomb repressive complex 1 (PRC1) compacts chromatin, causing the physical hinderance of RNA polymerase II, repressing gene transcription. PRC1 has E3 ligase activity, while polycomb repressive complex 2 (PRC2) has HMT activity. One of the best characterised PRC2 subunits is EZH2, which is involved in the methylation of lysine 27 on histone H3. PcG-target genes often contain both repressive (H3K27me3) and active (H3K4me3) modifications [22]. Thus, PcG-target genes exist in a poised ready-to-transcribe state with PcGs holding RNA polymerase II at the transcription start site [22].

HATs (e.g. p300/CBP) are classified into type A (nuclear) and type B (cytoplasmic), depending on whether they acetylate nucleosomal histones or newly translated non-nucleosomal histones, respectively. HATs transfer acetyl groups from acetyl-CoA donors to the amino group of lysine residues of histones. Acetylation of an ε-amino group neutralises the charge of lysine residues, reducing interactions between histones and DNA and making DNA less compact and more accessible to transcription factors. Histone acetylation is associated with gene activation, while deacetylation silences genes. Anti-cancer drugs that target HATs (HAT inhibitors) include the small molecule C646, which selectively inhibits p300/CBP, resulting in reduced acetylation of histone H3 [23]. C646 reduces cell survival and induces cell cycle arrest, mitotic catastrophe and apoptosis [23,24].

**Figure 4:**
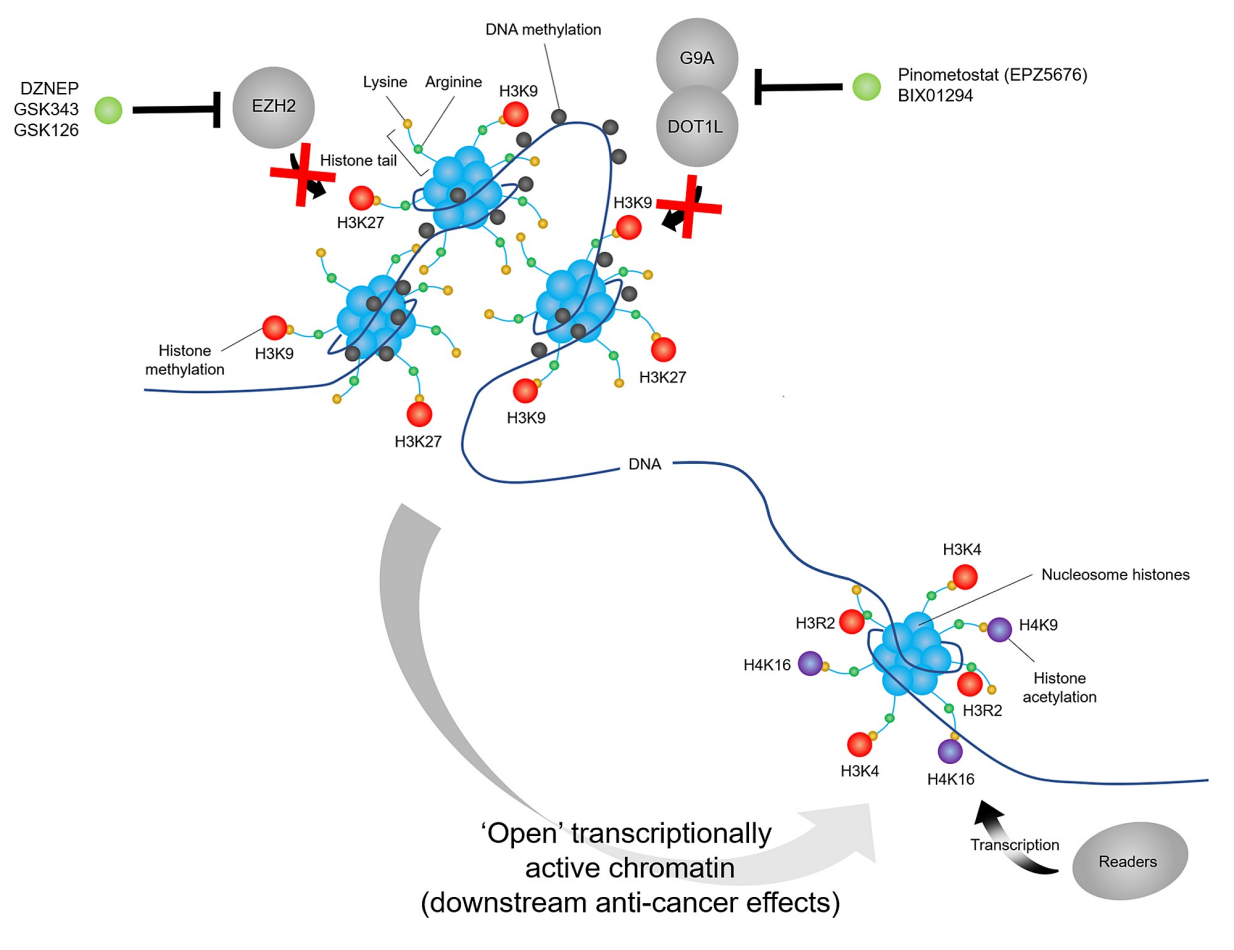
Mechanism of action of HMTIs HMTIs inhibit histone methylation by HMTs (e.g. EZH2, DOT1-like histone lysine methyltransferase [DOT1L], euchromatic histone lysine methyltransferase 2 [G9A]), leading to reduction of repressive histone marks (e.g. H3K9 and H3K27 methylation) and adoption of an ‘open’ transcriptionally active chromatin state with downstream anti-cancer effects. HMTIs, histone methyltransferase inhibitors; HMT, histone methyltransferase

Anti-cancer drugs (HMT inhibitors) have also been developed that target the HMTs EZH2, DOT1-like histone lysine methyltransferase (DOT1L), euchromatic histone lysine methyltransferase 2 (G9A), the histone-lysine N-methyltransferase SUV39H1 and protein arginine methyltransferases (PRMT1/3/45/6/8). The EZH2 inhibitor DZNEP reduces H3K27 trimethylation, leading to apoptosis and reduced cell migration (Figure 4) [25]. GSK343 induces apoptosis by increasing caspase-3 and poly ADP-ribose polymerase expression, induces autophagy by inhibiting expression of p62 and suppresses cancer stem cell-like phenotypes [26,27]. GSK126 reduces anti-tumour immunity by increasing the number of myeloid-derived suppressor cells, leading to fewer CD4+ and CD8+ T cells in the tumour microenvironment [28]. DOT1L inhibitors include pinometostat (EPZ5676), which reduces H3K9 methylation and produces a modest clinical response in a subset of adults with advanced acute leukaemia [29], although resistance does occur through drug efflux dependent (ABCB1) and independent mechanisms [30]. The G9A inhibitor BIX01294 inhibits proliferation by downregulating H3K9me1, H3K9me2, H3K27me1 and H3K27me2 modifications, downregulating the anti-apoptotic protein Bcl-2 and upregulating the pro-apoptotic proteins Bax, caspase-3 and caspase-9 [31]. Finally, the SUV39H1 inhibitor chaetocin increases production of reactive oxygen species (ROS), upregulates death-receptor genes and increases expression of CD11b, a surface integrin involved in adhesion interactions with immune cells [32,33].

**Figure 5:**
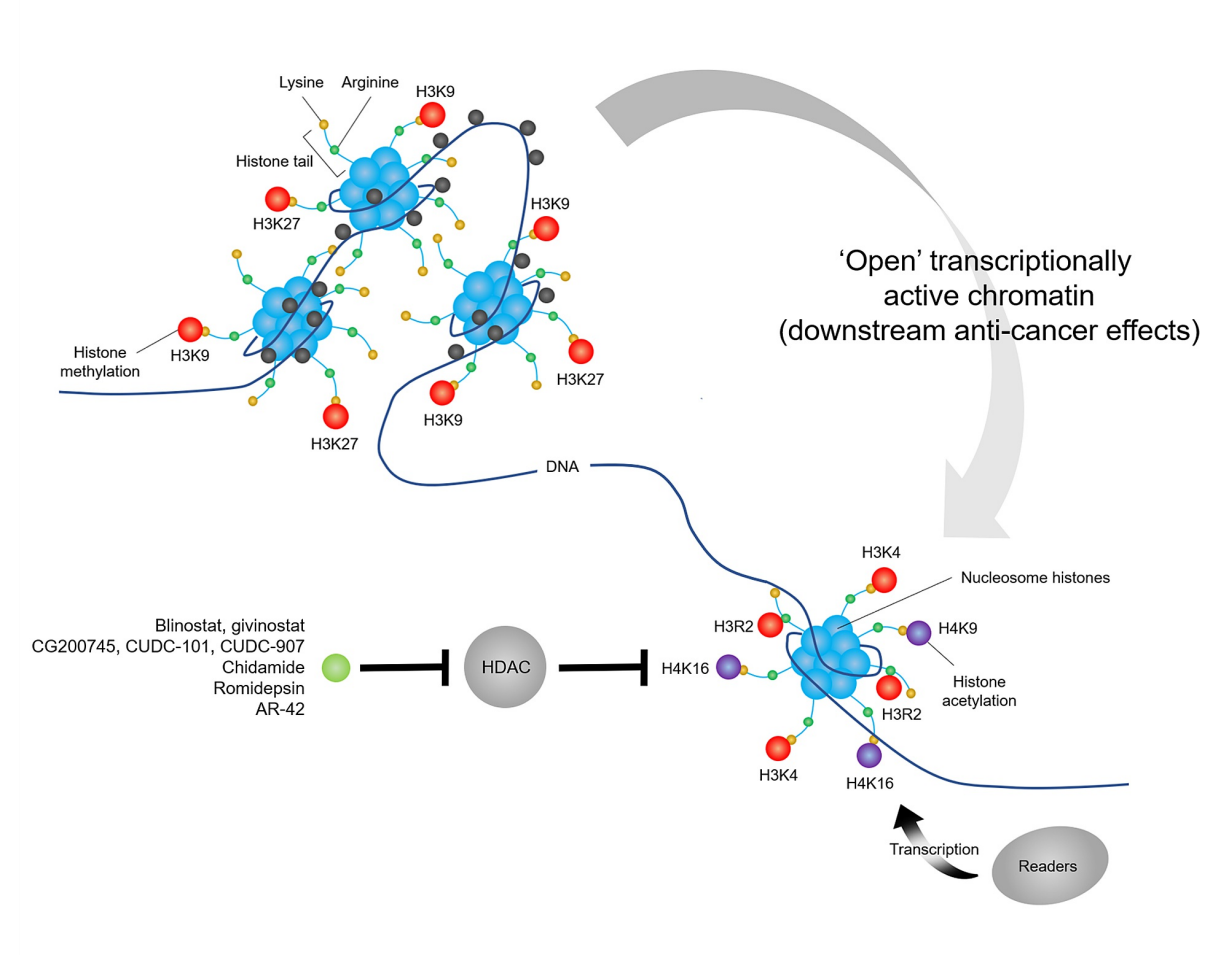
Mechanism of action of HDACIs HDACIs inhibit the deacetylation of histones by HDACs, leading to an increase in activating histone marks (e.g. H3K9 and H3K16 acetylation) and adoption of an ‘open’ transcriptionally active chromatin state with downstream anti-cancer effects. HDACIs, histone deacetylase inhibitors; HDAC, histone deacetylase

Erasers, such as HDACs and histone demethylases (HDMs), remove histone acetyl and methyl groups from histones. HDACs are classified as class I (HDAC1/2/3/8), II (HDAC4/5/6/7/9/10), III or IV. Classes I, II and IV are Zn2+-dependent, while class III is nicotinamide adenine dinucleotide (NAD)-dependent. Dysregulation of HDACs causes a global reduction in histone acetylation, which silences tumour suppressor genes. Anti-cancer drugs that target HDACs (HDAC inhibitors) include hydroxamates, benzamides, cyclic peptides and fatty acids; many of which target the Zn2+ ion in the HDAC active site. HDAC inhibitors reduce oncogene transcription and signalling, thereby promoting cell cycle arrest and apoptosis (Figure 5). Hydroxamate HDAC inhibitors (belinostat and givinostat) reduce cell survival by causing cell cycle arrest through the induction of p53, induce autophagy and inhibit stemness through the upregulation of differentiation markers (GFAP, Tuj-1) [34,35]. Novel hydroxamate HDAC inhibitors (CG200745, CUDC-101, CUDC-907) reduce cell survival by inducing apoptosis through the downregulation of Hippo pathway proteins and the upregulation of microRNAs (miR-210-3p, miR-509-3p), triggering caspase-dependent degradation of the promyelocytic leukaemia-retinoic acid receptor alpha (PML-RARA) fusion protein and apoptosis in a Mcl-1, Bim and c-Myc dependent manner [36-38]. The benzamide HDAC inhibitor chidamide induces mitochondrial dysfunction, necroptosis and apoptosis [39]. The cyclic peptide HDAC inhibitor romidepsin induces cell cycle arrest and apoptosis by increasing the acetylation of BCL6 [40]. Finally, the fatty acid HDAC inhibitor AR-42 induces cell cycle arrest and apoptosis by inhibiting the AKT/NFκB pathway [41]. Unlike DNMTIs, HDAC inhibitor monotherapy offers low clinical efficacy, achieving poor overall response in AML. However, combination regimens of HDAC inhibitors with DNMTIs, conventional chemotherapy or allogeneic stem cell transplantation have produced encouraging results. Notably, isozyme-selective HDAC inhibitors offer improved safety profiles and comparable efficacy to pan-HDAC agents [42].

HDM inhibitor anti-cancer drugs have been developed that target the HDMs lysine demethylase 1A (LSD1/KMD1A) and 5B (KDM5B) as well as JmjC domain-containing proteins. JmjC demethylases are protein hydroxylases involved in free radical-dependent histone modification reactions [43]. LSD1 inhibitors (ORY1001, GSK2879552, tranylcypromine) induce cell cycle arrest and apoptosis by regulating the hexokinase 2 expression and increase the expression of the transcriptional repressor GFI1 as well as the transcription factor PU.1, thus inducing differentiation [44,45]. However, a recent phase I open-label trial of the LSD1 inhibitor GSK2879552 was terminated early due to poor disease control and an unfavourable side-effect profile [46]. JmjC domain-containing protein inhibitors (GSK-J1, GSK-J4) induce apoptosis and inhibit tumour growth by increasing global levels of repressive trimethylated H3K27 and downregulating cancer-promoting HOX genes [47,48].

## Conclusions

Cancer epigenetics is a highly complex and rapidly evolving field, with many exciting developments that enhance our understanding of carcinogenesis and disease progression. By examining epigenetic networks, novel therapeutic approaches can be identified that encompass a wide variety of solid and haematological cancers. Therapeutics that normalise or disrupt epigenetic aberrations hold promise across several malignancies, with well-defined clinical efficacy established in distinct clinical settings. Considering the abundance of epigenetic targets and agents in development, a systematic approach for the identification and validation of potential drug targets is essential to optimise drug development and translate the promise of upcoming epigenetic agents to routine patient management. Future research should focus on achieving a deeper understanding of epigenetic mechanisms to yield better therapies as well as exploiting therapeutics that promote global epigenetic normalisation to counteract epigenetic aberrations. These approaches will enhance the utility of epigenetic drugs, maximising benefits in terms of returns of research investment and alleviating the burden of cancer on public health.
